# All-Male Groups in Asian Elephants: A Novel, Adaptive Social Strategy in Increasingly Anthropogenic Landscapes of Southern India

**DOI:** 10.1038/s41598-019-45130-1

**Published:** 2019-07-04

**Authors:** Nishant Srinivasaiah, Vinod Kumar, Srinivas Vaidyanathan, Raman Sukumar, Anindya Sinha

**Affiliations:** 1 National Institute of Advanced Studies, Animal Behaviour and Cognition Programme, Bengaluru, 560012 India; 2 Foundation for Ecological Research, Advocacy and Learning, Pondicherry, 605101 India; 3 Indian Institute of Science, Centre for Ecological Sciences, Bengaluru, 560012 India; 4 Indian Institute of Science Education and Research Kolkata, Mohanpur, 741246 India

**Keywords:** Behavioural ecology, Conservation biology

## Abstract

Male Asian elephants are known to adopt a high-risk high-gain foraging strategy by venturing into agricultural areas and feeding on nutritious crops in order to improve their reproductive fitness. We hypothesised that the high risks to survival posed by increasingly urbanising and often unpredictable production landscapes may necessitate the emergence of behavioural strategies that allow male elephants to persist in such landscapes. Using 1445 photographic records of 248 uniquely identified male Asian elephants over a 23-month period, we show that male Asian elephants display striking emergent behaviour, particularly the formation of stable, long-term all-male groups, typically in non-forested or human-modified and highly fragmented areas. They remained solitary or associated in mixed-sex groups, however, within forested habitats. These novel, large all-male associations, may constitute a unique life history strategy for male elephants in the high-risk but resource-rich production landscapes of southern India. This may be especially true for the adolescent males, which seemed to effectively improve their body condition by increasingly exploiting anthropogenic resources when in all-male groups. This observation further supports our hypothesis that such emergent behaviours are likely to constitute an adaptive strategy for male Asian elephants that may be forced to increasingly confront anthropogenically intrusive environments.

## Introduction

Social organization in mammals is defined by their group size and demographic composition and is known to be influenced by certain biological characteristics, including species-specific phylogenetic inertia and intrinsic individual-level attributes, such as age, sex, physiology or genetic traits. Additionally, environmental factors such as resources, human-driven-threats and other stochastic events are known to affect social organisation^1–8^. Increasingly, a number of mammalian species seem to be coping with large-scale changes in their environment mainly driven by anthropogenic factors through behavioural flexibility rather than commonly discussed genetic adaptations^5–7,9,10^. Such developmental and social flexibility in behaviour allow for the expression of alternative strategies that may enable certain mammalian populations to successfully survive and reproduce in changing environments^2,6^.

This is especially true in the case of elephants, which have well-defined growth phases^11,12^ and sex-specific life-history strategies (reviewed in^13^). Elephants live in mixed-sex social units termed as families, bond-groups or clans^13–16^. Changes in the sociality of male Asian elephants is pronounced between 11 and 20 years, as adolescent males disperse from their natal herds transitioning into socially mature adults. An important physiological change that occurs in male elephants during this phase is the onset of musth^17,18^, characterized by enhanced testosterone level and increased sexual activity^13,19^. When in musth, male African and Asian elephants are known to associate with females to mate with them^13,20,21^. Dynamic changes in social associations and behavioural strategies seem to be appearing amongst both female and male elephants, in populations that are living in rapidly changing ecological and anthropogenic environments^21–23^. For instance, male African elephants are known to associate in large all-male groups of highly related individuals of similar age^24^ in areas with high primary productivity and anthropogenic mortality risk^21^. Adult Asian elephant males are typically solitary but could also form small, short-term, all-male groups, especially during crop-foraging^18^.

Male Asian elephants are known to forage on agricultural crops nearly six times more, on average, than do female-led family groups in certain populations of southern India^25^. It has been proposed that such male elephants may be adopting a high-risk high-gain foraging strategy^25,26^ to improve their body condition and come into musth, thereby increasing their reproductive success^27^. As compared to protected forests, foraging in human-production habitats not only improves body condition, but also lowers nutritional stress in male elephants^28^. Such males, however, are highly prone to human-related stressors including injuries and deaths^29,30^. Human-elephant conflict-related deaths of elephants due to electrocution, poisoning, shooting and accidental deaths is high. In India alone, nearly 150 elephants succumb to human-elephant conflict every year^31^. The faecal glucocorticoid metabolite levels in solitary adult male elephants are known to increase significantly following antagonistic interactions with humans, such as elephant drives^32^. Such physiological stress levels are higher than that of adult females in herds in the same human-dominated landscape^32^, suggesting that social buffering (associating in groups) may effectively lower acute stress in elephants.

In this paper, we assess the relative influence of biological variables such as age and musth, and of environmental factors such as forest- or natural habitat contiguity as well as anthropogenic influences, such as changing landuse patterns and other human activities on the sociality of male Asian elephants in an agricultural landscape of southern India. With well-established body size and predictable musth periods, we expect very little flexibility in social decision-making in mature adults that mostly remain solitary. On the other hand, sexually mature but socially immature males in the adolescent stage are likely to display high inter-individual variability in sociality as they disperse from their natal herds to explore new environments and make important foraging- and social decisions that aid in their successful transitioning into socially mature adults. For mature male elephants that have dispersed from their natal groups, therefore, remaining solitary in areas with high human-activity may make them highly vulnerable to human-induced stress and prone to direct threats such as poaching and retaliatory killings^13^. Additionally, young dispersing males individually conduct trial-and-error exploratory forays to new habitats or resources^33^. Such explorations are occasionally maladaptive and may be costly, especially if they select high-risk landscapes of low productivity (reviewed in^8^). Hence, grouping together of males may evolve as a behavioural strategy to exploit resources while minimizing the per-capita risks in male elephants.

We, therefore, firstly hypothesised that the decision by male Asian elephants to associate in all-male groups in highly human-dominated areas may be driven primarily by environmental factors and anthropogenic influences rather than biological factors. Secondly, we hypothesised that the group size of all-male groups in high-risk agricultural habitats such as the crop-growing regions of Tumkur, Ramanagara and Krishnagiri districts will be larger than those in low-risk, forested habitats such as the Protected Areas of Bannerghatta National Park, Cauvery Wildlife Sanctuary and Cauvery North Wildlife Sanctuary (Karnataka and Tamil Nadu states, India). Finally, we hypothesised that associating in all-male groups may be an adaptive social behaviour, especially for the adolescent males, as foraging in resource-rich agricultural landscapes would enable them to improve body condition and become reproductively successful, large dominant bulls in the society.

## Results

During the study period (February 2016–December 2017) we sampled for a total of 10,705 days and obtained 20,124 photo-captures of elephants. From these elephant photographs, excluding calves, we were able to identify a total of 248 distinct males. Of these, 25 males were classified as Sexually and Socially Mature (SSM, above 20 years of age), 113 as Sexually Mature but Socially Immature (SM, from 10 to 20 years of age) and 110 as Sexually Immature (SIM, <10 years of age). Based on the frequencies of photographic records (n = 1445), SSM comprised nearly 20%, SM 43% and SIM 37% of the male elephant population in the study site. We thus observed relatively fewer old and mature bulls in the study population.

Male elephants in the intensive study area primarily occurred in mixed-sex groups (43.36%, n = 620), followed by solitary (33.57%, n = 480) and in all-male groups (23.08%, n = 330). These associations ranged in size from one (solitary) to 25 individuals. The mean (±SE) size of the mixed-sex groups was 8.53 (±0.17, range 1 to 25, n = 620) and that of all-male groups 3.59 (±0.12, range 2 to 12, n = 330). While SIM males were sighted mostly in mixed-sex groups, SM males were observed to be solitary or in all-male groups in equal proportions and the SSM males were seen to be mostly solitary (Supplementary Table 1**)**. A significant difference in the occurrence of the three maturity classes across the three different social units was thus observed (G-test of independence, G = 500.21, df = 4, p < 0.001). Hence, mixed-sex groups consisted mostly of juvenile and all-male groups mostly of adolescent males while matures males remained mostly solitary (Supplementary Table 2**)**.

### All-male associations

**Figure 1:**
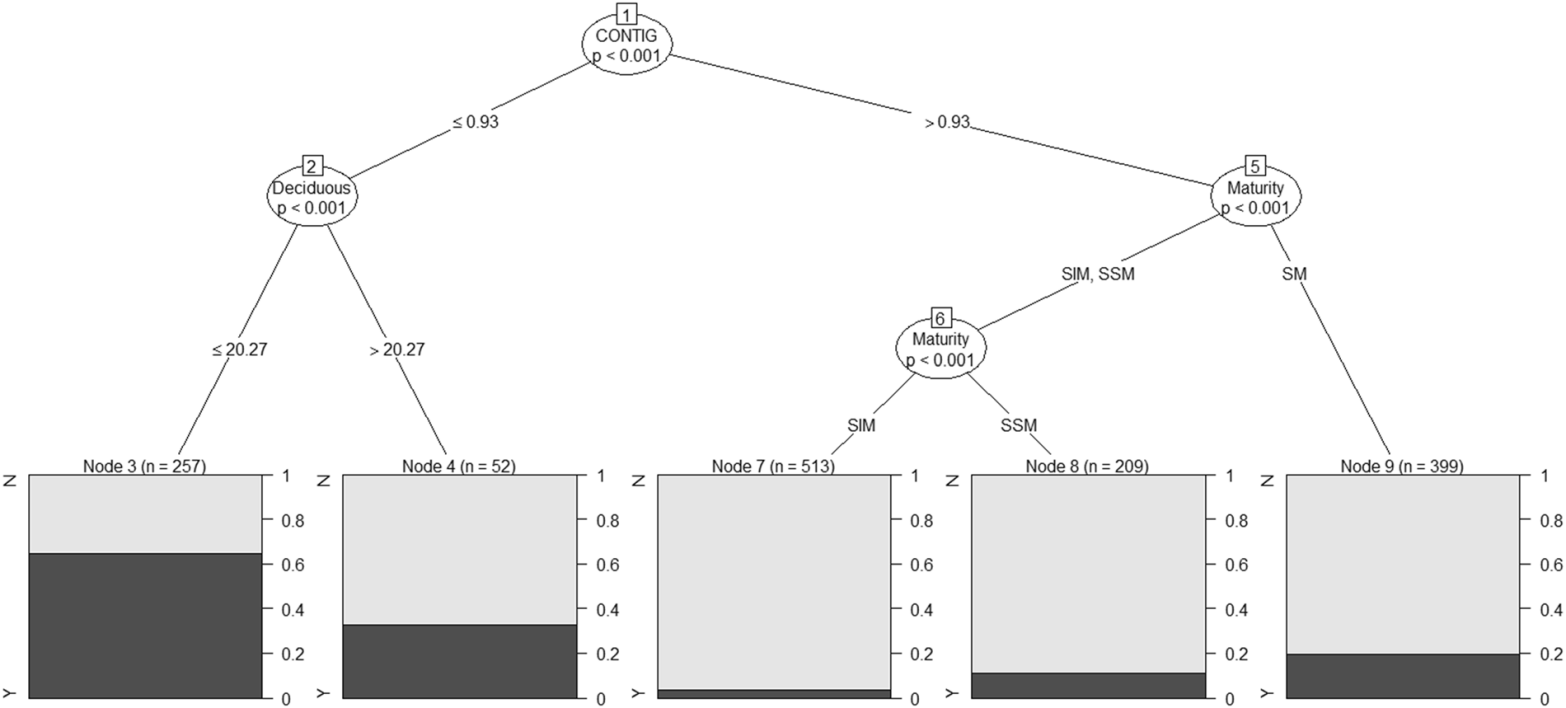
Classification tree showing the relative importance of the different biological and environmental attributes, with statistically significant branches at Nodes, in determining the propensity of male elephants to occur in all-male groups. SIM: Sexually Immature, SM: Sexually Mature but Socially Immature, SSM: Sexually and Socially Mature, A: Musth absent, P: Musth present, Y: Associating in all-male groups, N: Not associating in all-male groups.

The primary factor determining the association of males in all-male groups was Forest Contiguity (G = 108.08, df = 1, p < 0.001, Fig. 1; Node 1). Nearly 65% (166 of 257, Fig. 1; Node 3) of male elephant sightings in all-male groups were in areas with Forest Contiguity (CONTIG) ≤ 0.93 and Deciduous Forest ≤ 20.27%. This was not significantly different from their propensity to occur in all-male groups in CONTIG ≤ 0.93 areas with Deciduous Forest > 20.27% (G = 2.00, df = 1, p = 0.157). A significant difference in the association of male elephants across the three maturity classes was also observed in areas with CONITG > 0.93 (G = 26.22, df = 2, p < 0.001). The SM males showed the highest propensity of association in all-male groups (19.54%, Node 9) followed by SSM males (11.50%, Node 8) while the SIM males displayed the lowest propensity at 3.31% (Node 7). Thus forest contiguity was the most significant factor determining all-male group formation in Asian elephants with SM males showing the highest propensity to associate in such groups.

### Mixed-sex associations

Maturity was the first and the most significant factor determining the association of male elephants in mixed-sex groups (G = 117.45, df = 2, p < 0.001, Supplementary Fig. 1; Node 1). A relatively high proportion (nearly 85%) of SIM male sightings were in areas with Crop ≤ 39.17% and Human Use Index (HUI) ≤ 3.1 (Supplementary Fig. 1; Node 4). The SM males showed highest propensity to associate in mixed-sex groups (56%) in areas with CONTIG > 0.93 and Degraded Forest > 2.95. The SSM males in musth showed a significantly higher propensity to associate with mixed-sex groups (72.72%) than when not in musth (G = 4.55, df = 1, p = 0.03; Supplementary Fig. 1). The maturity of the individual was thus the primary factor influencing decision-making in male elephants that associated in mixed-sex groups, with SIM males showing highest propensity to associate in such groups followed by SSM males in *musth*.

### Solitary males

Maturity was once again the primary determinant of the occurrence of male elephants as solitary individuals in the intensive study area (G = 70.27, df = 2, p < 0.001, Supplementary Fig. 2; Node 1). SSM males were observed to be solitary at a relatively high proportion of 62.06% (Node 10) in Deciduous Forest areas. Their occurrence in areas with Deciduous Forest >37.55% was not significantly different than observed in areas with less Deciduous Forest (G = 1,20, df = 1, p = 0.273, Node 11). For SM males, the propensity to remain solitary was significantly higher (53.15%) in areas with Crop ≤ 9.28 than in areas with more Crop (G = 4.27, df = 1, p = 0.04). SIM males on the other hand remained mostly solitary in areas with Crop > 44.85% (n = 13, Supplementary Fig. 2; Node 6). The maturity of the individual was thus the primary factor influencing decision-making in male elephants that occurred solitarily, with male elephants tending to become increasingly solitary with age, but mainly in Deciduous Forest.

### Social group size in all-male groups

**Figure 2:**
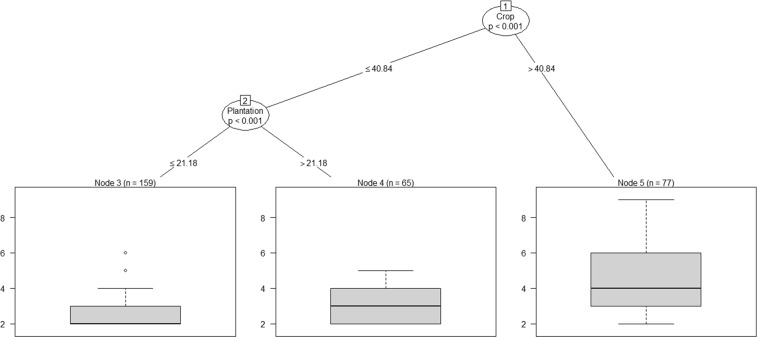
Classification tree showing the relative importance of the different biological and environmental attributes, with statistically significant branches at Nodes, in determining the size of all-male groups of elephants.

There was a significant variability in the size of all-male groups in the intensive study area (Fig. 2). In cells with Crop > 40.8%, the group size was the highest, with a mean (±SE) of 4.64 (±0.27, range 2 to 9, Node 5). In areas with Crop < 40.8% and Deciduous Forest ≤ 20.3%, group size reduced to a mean of 3.13 (±0.12, range 2 to 6, Node 4). The size of all-male groups, however, was the least in areas with Crop ≤ 40.8% and Deciduous Forest > 20.3%, with a mean of 2.29 (±0.06, range 2 to 4, Node 3). The size of all-male groups was thus the highest in areas under intensive cultivation and low deciduous forest.

### Body Condition of males in all-male groups

There was a significant difference between the Body Condition (scored on a scale of I to V) of males in all-male groups (G = 16.68, df = 3, p < 0.001), mixed-sex groups (G = 35.91, df = 3, p < 0.001) and those that remained solitary (G = 13.23, df = 3, p < 0.004). Nearly 58% of males in all-male groups, 54% males ranging solitary and only 27% males in mixed-sex groups were scored V on Body Condition (Supplementary Table 3). None of the males in all-male groups were assigned a Body Condition score below IV.

**Figure 3:**
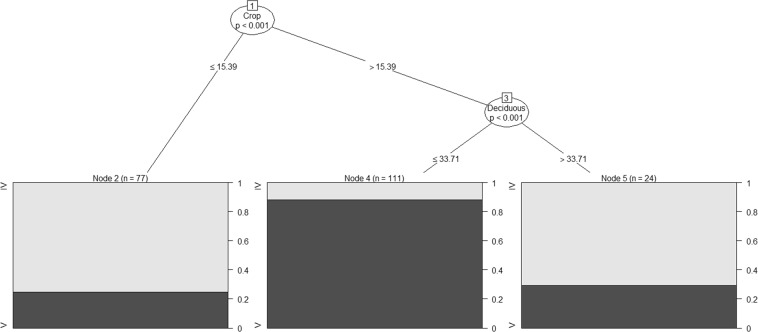
Classification tree showing the relative importance of the different biological and environmental attributes, with statistically significant branches at Nodes, in determining the Body Condition score, on a scale of I to V, of SM males in all-male groups. SM: Sexually Mature but Socially Immature.

On examining the Body Condition of males in all-male groups specifically, we found that only the Body Condition of SM males was influenced by availability of Crop and Deciduous Forest (Fig. 3), while both SSM and SIM males were not influenced by any of the select biological and environmental factors (Supplementary Figs 1 and 4). A significant difference in the Body Condition of SM males in areas with Crop > 15.39% was observed when compared to areas with less Crop (G = 11.70, df = 1, p < 0.001, Fig. 3; Node 1).

## Discussion

In this study, we report for the first time, the occurrence of large and stable all-male groups and the processes that drive the formation of these novel and long-term associations in male Asian elephants. We discuss these findings with respect to our current understanding of elephant sociality and life-history strategies with a special emphasis on the evolving behavioural adaptations in male Asian elephants that are increasingly using human-dominated landscapes. We further discuss the implications of these adaptations with respect to the management and conservation of Asian elephant populations.

Male elephants are typically born into a matriarchal natal group and continue to stay in it at least until adolescence^18^. Accordingly, we too found sexually and socially immature (SIM) males to predominantly occur in mixed-sex groups in our study area. In Asian elephant society, it is well established that male elephants on reaching adolescence disperse from their natal groups in search of unrelated females to mate with and forage-rich areas in which to establish themselves^18^. In the study population SM males and on very few occasions even older SIM males showed a tendency towards such behaviour by ranging in cropfields solitarily, however, only for a short-duration (<12 h). For a number of sexually mature but socially immature (SM) dispersing males, movement into unknown habitats in search of food, water and other elephants, may become maladaptive, due to a lack of knowledge of the risks that the newly occupied areas pose^34^. This is especially true in fragmented human-modified production landscapes, which typically characterized our study region. In our study, we observed that adolescent male elephants associated in large bull groups almost exclusively in human-modified landscapes, predominantly with croplands but interspersed by fragmented and isolated forest patches, supporting our first hypothesis that the recent all-male group formations in Asian elephants may be environmentally rather than biologically influenced.

Our study elephants occurred in a region close to major towns and cities, such as Bangalore, also known as the Silicon Valley of India. This region has undergone major landuse changes, especially between the years 1973 and 1992, with rapid increase in agriculture, human densities, major and minor roads and a concomitant burgeoning of the urban sprawl, all at the expense of forest cover and natural elephant habitats^35,36^. Reforestation, in the form of monoculture of exotic tree species such as *Acacia auriculiformis* and *Eucalyptus* spp., in the years between 1992 and 2007, following deforestation, mainly outside Protected Areas^36^ (PAs) has resulted in the reduction in cultivation of subsistence crops bordering PAs, which may have increased tree cover for elephants, but may not have helped them nutritionally. More recently, quarrying activity in hillocks adjoining the PAs has resulted in the further loss of natural habitats and caused increased disturbance to elephants.

These rapid and large-scale changes in landuse, within a lifetime of an individual elephant, may have provided a perfect setting in which elephants need to be behaviourally flexible and adapt to these dynamic and potentially risky production landscapes. Conflict-related injuries and mortality were recorded in our study area too with eight adolescent and two mature adult males succumbing to such injuries or captures in 15 months within the study period. Given such environmental stochasticity, associations of male elephants may have emerged as a behavioural necessity for young male elephants in high-risk, high-resource landscapes, especially in recent years (see also^33,37,38^).

The all-male groups increased in size with increasing crop availability, supporting our second hypothesis that the group size of such associations would increase in resource-rich, high-risk areas. These findings thus suggest that available environmental resources and certain anthropogenic factors may play a more significant role, than would intrinsic factors, in shaping the social decisions of males associating in all-male groups, similar to their African counterparts^21^. The relatively high intraspecific variability in social organization displayed by SM males and their increased propensity to associate with other males of the same or older age classes is reminiscent of what has been observed in African elephant populations as well^12,24^. It is noteworthy that studies conducted on elephants in our study region, more than two decades ago, do not mention the occurrence of these large and stable all-male groups^35^. One of us (RS) has been observing social groupings of elephants in the larger study landscape since the 1980s and began to observe such large all-male associations in the human-use areas approximately over the last two decades. This suggests that this type of association of elephants may be of a rather recent origin in this landscape.

We found the male elephants in our study population, especially the SM males that used production landscapes, to have significantly better body condition than those inhabiting areas with relatively more deciduous forest, supporting our third and final hypothesis that all-male group formation could be adaptive. Foraging on crops may, therefore, be an effective strategy for these young dispersing males to increase their body size relatively rapidly.

For the sexually and socially mature (SSM) males too this strategy may serve to maintain good body condition enabling them to stay in musth for longer periods of time (Srinivasaiah N. M., Sinha, A., Vaidyanathan, S. & Sukumar, R. unpublished data). When in musth, these males either moved solitarily in search of estrus females or associated with mixed-sex groups in deciduous forest areas, possibly to increase their chances of mating. We also found SSM males when not in *musth* to remain largely solitary in forested habitats, which conforms with previous studies on Asian elephants^13^. Hence, biological rather than environmental attributes influenced group formation in the case of SSM males.

Small- and medium-sized all-male groups, with a high turn-over between particularly associating individuals, were observed either within the large forested habitats or in cropfields adjacent to such habitats alone. These short-term associations (for a few weeks in a year) of male elephants, were those of either peer group members or a cohort, usually of older SIM (5 to 10 years of age) or SM individuals, formed in response to a *musth* bull, which may have associated with their natal herd and chased the younger males away. Such a social trigger may also be the first step towards dispersal in young male elephants. The medium-sized all-male groups were again short-term associations (for one cropping season in a year) of males of the SM and SSM class. Such associations, were observed mostly along the fringes of forests and nearby cropfields, and has been well-documented in previous studies^18^. Finally, the large all-male groups that were observed in highly-fragmented, predominantly agricultural areas were long-term (for a few years or more) associations of males mostly belonging to the SM class and which is of particular interest to this study. It is important to note that we have observed some of these large all-male associations of SM males to consist of particular individuals, who have been stably associating with one another for over ten years now, since our long-term study was initiated. While the variation in group size in mixed-sex groups of elephants have been well established in the study area (in press^39^), our observations on intraspecific variation in social organisation of male Asian elephants indicate that the emergence of large, stable all-male groups in response to extrinsic environmental factors is a rather novel phenomenon. We would also like to point out that such a social strategy adopted by our study male elephants may represent a specific example of more general risk-management strategies increasingly being displayed by elephant populations across their threatened habitats.

Elephants are thus not unusual in coping with increasing anthropogenic pressures in their changing environments by displaying significant social flexibility, as has been shown in other mammalian species as well (reviewed in^6,7^). The behavioural flexibility, primarily shown by young male elephants in the dispersal stage and manifest through the formation of all-male groups and adoption of novel foraging strategies, leading to improved body condition, may thus constitute an example of how even large mammals such as elephants can develop behavioural strategies to increase their survival and reproductive fitness^7,8^.

The mortality of elephants, due to conflict with local human communities and the various resultant interventions, however, is an enormous conservation challenge^40^, especially for males in already male-depleted populations^41^. The lack of mature adult males in such populations, which have also endured high poaching rates in the past^13,18^, may lead to reduced population growth rates^42^ and may have behavioural implications for younger individuals who then grow up in an environment without role models to learn from^43^. Young male elephants in the study landscape are known to associate with older males in human-use areas^23^. Under these circumstances, losing even more males through highly intrusive conflict mitigation measures, such as captures or retaliatory killings, could have a further negative effect on populations, in a manner like to that of poaching.

It may therefore be imperative that future attention is focused on the management and conservation of young dispersing males of this highly endangered species, as the often-flexible decisions made by these individuals appear to directly influence the utilization of production landscapes by the species, thus bringing them into direct conflict with local agricultural communities. An understanding of such emergent behaviours in elephants may also provide us with strategies to reduce human-elephant conflict while keeping in mind the sociality of Asian elephants. Although the management and conservation strategies for the increasingly threatened Asian elephant populations need to be explored in the near future, we have only now begun to understand the more fundamental aspects of this newly emergent social organization in male Asian elephants in human-modified landscapes.

## Methods

### Study area

**Figure 4:**
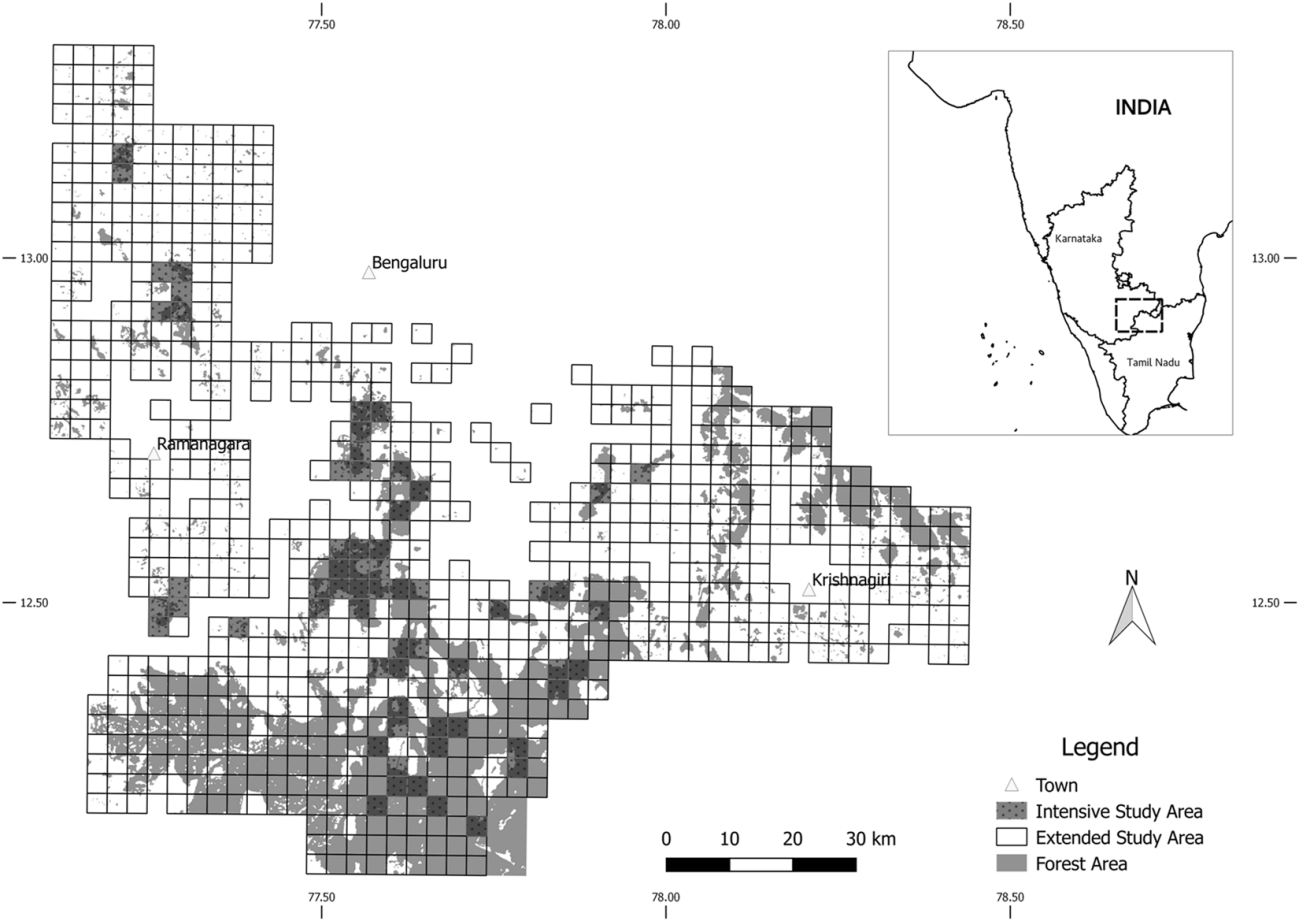
Map of the study area in southern India showing a matrix of forested and non-forested habitats of the study elephant population, the survey area and the intensive study area.

Our prior knowledge of elephant ranging patterns over a nine-year period since 2009, acquired through following individual elephants, allowed us to demarcate an extended study area of c. 10,000 km^2^, in southern India. This region was then overlaid with a grid, consisting of 1,000 cells of size 10 km^2^ each, using Quantum Geographical Information System^44^. Of these, 778 cells had forest patches within them and using a questionnaire survey, administered to experienced forest guards, we established the presence of elephants at least for six months annually in 118 of these cells (Fig. 4). These cells were then systematically surveyed by a team, comprising the researchers and experienced forest staff, for elephant presence at select waterholes, either through direct sightings or by indirect signs such as dung, tracks or evidence of foraging, over a period of one year, from February 2015 to February 2016, taking care that each cell was surveyed at least once every month. This initial survey also allowed us to determine that only 63 of these 118 cells, comprising c. 630 km^2^ were logistically amenable for long-term monitoring of elephants using the camera-trap method and was designated as the intensive study area (Fig. 4).

The extended study area is a matrix of human habitations, tropical deciduous forests and cropfields, extending over an area of c. 10,000 km^2^ and with an approximate human density of 255 to 431 persons/km^2^ ^45^, within the administrative boundaries of Karnataka and Tamil Nadu states in southern India. The forested area of c. 1000 km^2^, included the Protected Areas of Bannerghatta National Park and parts of Cauvery and Cauvery North Wildlife Sanctuaries, with a combined elephant density of around 1/km^2^ ^46,47^, as also the Reserved Forests of Ramanagara, Bengaluru and Hosur Forest Divisions (Fig. 4). The vegetation is mostly tropical dry deciduous and scrub woodland forests, with numerous riparian patches. The elevation ranges from 250 to 1,000 m above msl, sloping from north to south. The region records a mean monthly maximum and minimum temperatures of 35 °C (April) and 18 °C (January) respectively, and receives an annual rainfall of c.800 mm, with a mean monthly maximum and minimum of 170 mm (October) and 5 mm (January) respectively^45^. Agriculture is the major occupation in the region, with June to December comprising the main cultivation season. Agriculture is mostly rain-fed, with the region receiving both the southwest (summer) and northeast (winter) monsoons. The main crops grown were finger millet *Eleusine coracana* and maize *Zea mays*^45^. However, in well irrigated areas, paddy *Oryza sativa* cultivation was prevalent. In addition, plantation crops such as mango *Mangifera indica*, coconut *Cocus nucifera*, sugarcane *Saccharum officinale* and banana *Musa* spp. are cultivated alongside agro-forestry crops such as acacia *Acacia auriculiformi*s and eucalyptus *Eucalyptus* spp. The landscape is dotted with numerous artificial waterbodies and quarries and highly fragmented by road and rail networks. Human-elephant conflict was prevalent across the region with almost daily crop-foraging by elephants and ten human- and elephant lives each being lost during the study period of 15-month duration. A record high level of human-elephant conflict, was reported from this landscape, in the years 2005 and 2006^46^.

### Collection and analysis of photographic data

A single camera trap was placed within each of these 63 cells, at waterholes with elephant usage established through the initial survey (Fig. 4). Information on elephant age, sex, group size, group composition and musth state was collected and individual identification carried out, simultaneously across these locations using photographs obtained^48–51^. These cameras covered a gradient of forest fragmentation and human use within the study area (Srinivasaiah N.M., Sinha, A., Vaidyanathan, S. & Sukumar, R. unpublished data) and were operational both during the day and at night, ensuring spatio-temporal coverage. Systematic camera trapping for elephants was conducted in the intensive study area between February 2016 and December 2017 for 10,705 days of 24-h period each (mean ± SE of 169.92 ± 18.94 days/camera), resulting in a total of 20,124 records. A comparable effort was invested in camera-trapping across a gradient of fragmentation within the intensive study area with 142 trap-days/nights in the highly fragmented regions (CONTIG 0.63 to 0.95), 185 trap-days/nights (CONTIG 0.95 to 0.97) and 153 trap-days/nights (CONTIG 0.97 to 0.98) on an average. We selected any one sighting of an individual elephant or a group, as defined below, from all operational cameras within a 12-h period for analysis; this resulted in a sample size of 1445 independent records (mean ± SE of 5.80 ± 0.57 sightings/individual, range from 1 to 53). Accurate recordings of group size were not possible on five of these occasions and of musth status on ten occasions. Hence, the total sample size to assess the impact of biological and environmental influences on the social organization of the study male elephants was reduced from 1445 to 1430 records.

A group, comprising both male and female elephants, was considered as a mixed-sex group, one with only males an all-male group and single male elephants as solitary individuals. It may be noted here that, in general, the study elephant groups were periodically observed over space and time across our study area. More specifically, the all-male groups could be stably identified through the regular association of individuals, as identified by systematic camera-trapping over the study area, as described above.

A group was defined using a temporal measure. An elephant photographed within a three-hour interval of another individual at the same camera location was considered to be part of the same group. This was based on our observation that typically, all individuals of a known group arrived at specific waterholes between two and three hours of any of its members visiting the same. Moreover, our earlier observations, during this long-term study^23^, indicated that if behavioural observations were conducted within a single sampling session of three hours, there was no change in group membership within this period of time.

The number of elephants within a group was counted based on the identification of individuals by a number of morphological features^14,52^, as recognised in their camera-trap images obtained both diurnally and nocturnally (Supplementary Fig. 10). From the inception of the study, every individual elephant was identified either through direct observations or camera-trap photographs, all its visible morphological features recorded and a database of all the identified individuals in the study area created. Every subsequently recorded individual was compared with this database and an algorithm used to estimate the probability of match of the newly sighted elephant with the known elephants in the database. The top 10 matches were then manually compared independently by two observers to arrive at the identity of the individual. If there was no final match, the individual was entered into the database as a new elephant.

The age of an elephant was estimated based on relative shoulder height measures^18,52^, which is the standard surrogate measure for age estimation in wild elephants. This method was used to classify our study elephants using camera trap images, into five age categories namely, Calf (birth to 1 years of age), Juvenile (1 to 5 years), Subadult Stage One (5 to 10 years), Subadult Stage Two (10 to 15 years) and Adult (>15 years). It should be noted that the shoulder height of a 15 to 20 year-old male is comparable to that of a fully grown adult female^52^. Beyond 20–25 years of age, the shoulder height of elephants is known to asymptote and hence, other morphological features such as degree of folding and depigmentation of the ears, temporal and buccal cavity depression and prominence of domes^53^.

Our observations of male elephants in the study area along with other such studies on Asian elephant socioecology suggests that male Asian elephants do not express periodic and long *musth* periods at least until about the age above 20 to 25 years^13,17^, similar to African elephants^19^. The forays of SIM males away from their natal herds too started from around the age of 10 years and increased in frequency after the 10th year^13^. Hence, it was essential to categorise elephants using the maturity classes that we have, as it provides adequate importance to males in the adolescent age class (10 to 20 years of age), which have seldom been studied. Our repeated observations of wild male elephants and of those in captivity allowed us to establish a strong correlation between the age categories of male elephants and their maturity classes, namely, SIM, SM and SSM. Thus, individual males from 1 to 10 years constituted SIM males, those from 10 to 20 years SM males and those beyond 20 years SSM males.

The scoring of Body Condition of the study male elephants was carried out based on the prominence in visibility of an elephant’s backbone, ribs, shoulder- and pelvic bones (Supplementary Fig. 8). The elephants were scored on a scale of I to V^54^ (Supplementary Table 3). Musth in male elephants was noted based on the different stages of secretion of musth fluid from the temporal glands and urine dribbling^18,55–57^.

To assess the level of human- and associated activities such as livestock grazing at each camera-trap location, we developed a Human Use Index or HUI (modified from^58–61^), defined as the number of photographs of humans/livestock within one hour of one another, considered as a single event (Table 1).

**Table 1:** Contiguity Index and Human Use Index of the intensive study area.

Patch Characteristic	Mean Value	Minimum Value	Maximum Value	Standard Deviation	Standard Error
Contiguity Index	0.94	0.63	0.98	0.07	0.00
Human Use Index	1.39	0	7.98	1.67	0.04

### Landuse and other habitat characteristics

The study landscape was classified into different landuse types, based on geospatial data obtained from the National Remote Sensing Agency of the Government of India (downloaded from http://bhuvan3.nrsc.gov.in/cgi-bin/LULC250K.exe). The original 19 Landuse and Landcover (LULC) categories^62^ were merged to derive eight LULC categories: Deciduous Forest, Degraded Forest, Plantation (orchards), Crop (seasonal and multicrop), Current Fallow, Wasteland, Waterbody and Built-Up Area (Table 2). For the 63 cells in the intensive study area, selected as described above, we estimated the percentage of different LULC categories (Table 2). To estimate the contiguity of forests within the study area, we developed a Contiguity Index (CONTIG), using FRAGSTATS^63^, by a 3 × 3 moving window on a layer containing forest-only patches (adapted from^64,65^). The Contiguity Index layer assigned larger values to large contiguous forest patches across cells and smaller values to small isolated patches (Table 1), which resulted in a measure of spatial connectedness of forested patches.

**Table 2:** Landuse characteristics of the intensive study area.

Cell Characteristic	Mean Percentage	Minimum Percentage	Maximum Percentage	Standard Deviation	Standard Error
Deciduous Forest	57.91	3.36	99.95	30.66	0.81
Degraded Forest	2.55	0	12.28	3.12	0.08
Wasteland	10.13	0	57.51	12.42	0.33
Crop	17.79	0	67.30	15.50	0.41
Plantation	7.60	0	64.83	13.52	0.36
Waterbody	0.49	0	4.91	0.76	0.02
Built-Up Area	1.71	0	22.74	4.90	0.13
Current Fallow	1.82	0	26.41	4.20	0.11

The intensive study area, used extensively by elephants, primarily comprised Deciduous Forest, although it also included significant proportions of human-modified landuse, such as Crop, Plantation and Wasteland (Table 2).There was also a significant gradient of the Human Use Index across the study area, a reflection of the simultaneous use of these habitats by the local human communities (Table 1). Fragmented deciduous forests and cropfields were thus the predominant landuse types in the study area.

### Statistical analysis

To examine the influence of biological and environmental factors on the decision by males to associate in particular social group types, we constructed recursive partitioning classification trees in R, version 3.4.023^66^. We used classification trees as it allows for intuitive visualization of the results obtained from a dataset having both categorical and continuous variables^67–69^. They also aid in the stepwise, hierarchical expression of the relative importance of the different variables investigated. The ten input variables included two biological parameters, Maturity and Musth, and eight environmental parameters, namely Deciduous Forest, Degraded Forest, Wasteland, Crop, Plantation, Waterbody, Human Use Index (HUI) and Contiguity Index (CONTIG). The response variables measured were Group Size, Social Group Type, and Body Condition. Two of the landuse types, Built-Up Area and Current Fallow, were not used in the final analysis, as they did not offer any resource to the study elephants. We assessed the statistical significance of the differences in the propensity of occurrence of male Asian elephants in the three social group types, referred to above, as response to varying levels of the above biological and environmental parameters using multiplicity-adjusted Monte-Carlo simulated (n = 9999) p-values^66^. The G-test of independence was used to assess differences in the occurrence of different classes of males in the population, the demographic composition of associations and also as a post-hoc procedure to test for the statistical significance of the recursive partitioning classification trees obtained above^70^.

The Research Ethics Committee of our host institution, the National Institute of Advanced Studies (NIAS) in Bangalore, approved all the natural observation and camera trapping protocols under the NIAS Research Ethics Policy. Permission to conduct the natural observations on the study elephants was obtained from the Principal Chief Conservators of Forests (Wildlife) and Chief Wildlife Warden of the Karnataka and Tamil Nadu Forest Departments (permit number: PCCF(WL)/E2/CR-103/2013-14 and WL5(A)/21591/67/2015).

## Supplementary information

Supplementary Information

Supplementary Video 1

## Data Availability

The datasets generated during and/or analysed during the current study are available in the Dryad digital repository, [10.5061/dryad.957f2tv].
