# Supplementary material for: All-Male Groups in Asian Elephants: A Novel, Adaptive Social Strategy in Increasingly Anthropogenic Landscapes of Southern India
Source: Sci Rep. 2019 Jul 4;9:8678. doi: 10.1038/s41598-019-45130-1 (PMC6609637; doi:10.1038/s41598-019-45130-1)
Supplement: Supplementary file 1 — Supplementary Information [file 41598_2019_45130_MOESM1_ESM.pdf]

Supplementary Information

All-Male Groups in Asian Elephants: A Novel, Adaptive Social Strategy in Increasingly Anthropogenic Landscapes of Southern India

Authors: Nishant Srinivasaiah[1,*], Vinod Kumar[2], Srinivas Vaidyanathan[2], Raman Sukumar[3], Anindya Sinha[1,4]

Authors' Institutional Affiliations

[1]National Institute of Advanced Studies, Animal Behaviour and Cognition Programme, Bengaluru, 560012, India

[2]Foundation for Ecological Research, Advocacy and Learning, Pondicherry, 605101, India

[3]Indian Institute of Science, Centre for Ecological Sciences, Bengaluru, 560012, India

[4]Indian Institute of Science Education and Research Kolkata, Mohanpur, 741246, India

[*]Corresponding author:

Nishant Srinivasaiah

Mobile: +91 9741037793

Email: msnishant@gmail.com

All-Male Groups in Asian Elephants: A Novel, Adaptive Social Strategy in Increasingly Anthropogenic Landscapes of Southern India

FIGURE LEGENDS

Supplementary Figure 1

Classification tree showing the relative importance of the different biological and environmental attributes, with statistically significant branches at Nodes, in determining the propensity of male elephants to occur in mixed sex groups. SIM: Sexually Immature, SM: Sexually Mature but Socially Immature, SSM: Sexually and Socially Mature, A: Musth absent, P: Musth present,Y: Associating in mixed-sex groups, N: Not associating in mixed-sex groups.

Supplementary Figure 2

Classification tree showing the relative importance of the different biological and environmental attributes, with statistically significant branches at Nodes, in determining the propensity of male elephants to be solitary. SIM: Sexually Immature, SM: Sexually Mature but Socially Immature, SSM: Sexually and Socially Mature, Y: Solitary, N: Not solitary.

Supplementary Figure 3

Classification tree showing the relative importance of the different biological and environmental attributes, with statistically significant branches at Nodes, in determining the Body Condition score, on a scale of I to V, of SIM males in all-male groups. SIM: Sexually Immature, SM: Sexually Mature but Socially Immature, SSM: Sexually and Socially Mature.

Supplementary Figure 4

Classification tree showing the relative importance of the different biological and environmental attributes, with statistically significant branches at Nodes, in determining the Body Condition score, on a scale of I to V, of SSM males in all-male groups. SSM: Sexually and Socially Mature.

Supplementary Figure 5

Night-image of a tuskless SSM male in *musth*. Note the *musth* secretion from the temporal gland.

Supplementary Figure 6

Night-image of a tuskless SSM male in *musth*, with the left hind leg in full view with urine dribble stain.

Supplementary Figure 7

A full-frame day-image of an SSM male in *musth* (see the profuse temporal flow of musth secretion) used for individual identification as well.

Supplementary Figure 8

Lateral profile image of an SSM male in the study area used for identification (shape of tusks and nicks, and cuts in the right ear) and BCI scoring

Supplementary Figure 9

Frontal profile image of an SSM male, same individual as in the above photograph, in *musth* with urine dribble stain on the hind legs.

Supplementary Figure 10

Typical camera-trap images, in this case of VKT, a SSM male, that were used for individual identification. Note that the shape of the tusks, degree of ear folds, presence of ear nicks, and appearance of the well-formed domes were some of the characteristic features that facilitated the recognition of our individual study elephants.

Supplementary Video 1

All-male group of three male elephants including a tuskless male moving towards a waterhole in the medium-conitguity region of the study landscape

TABLES AND TABLE LEGENDS

Supplementary Table 1

Percentage occurrence of the different maturity classes in the social group types

Maturity Class	Percentage in All-Male Group	Percentage in Mixed-Sex Group	Percentage as Solitary
Sexually Immature (SIM)	5.45	80.64	13.91
Sexually Mature but Socially Immature (SM)	34.42	28.08	37.50
Sexually and Socially Mature (SSM)	21.28	16.67	62.06

Supplementary Table 2

Demographic composition of elephant social group types in the study area

Social Group Type	Percentage of SIM	Percentage of SM	Percentage of SSM
All-male Group	9.63	70.43	19.93
Mixed-Sex Group	66.10	26.66	7.24
Solitary	15.42	48.13	36.46

Supplementary Table 3

Distribution of Body Condition scores of male elephants in social group types

Social Group Type	Percentage Score II	Percentage Score III	Percentage Score IV	Percentage Score V
All-male Group	0	0	48.16	58.14
Mixed-Sex Group	0.15	0.92	71.96	26.96
Solitary	0.21	0.42	45	53.38

FIGURES

Supplementary Figure 1

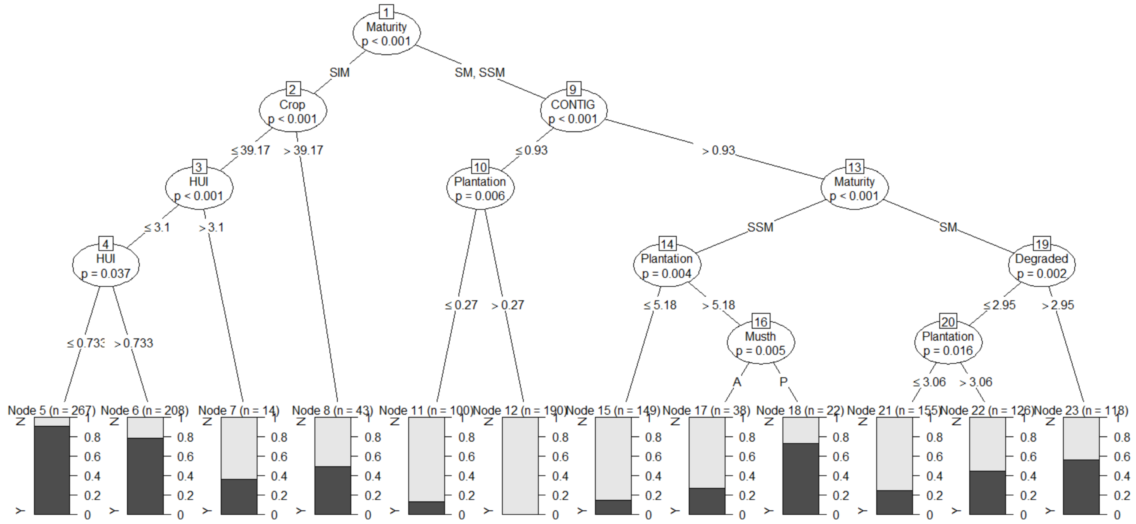

Supplementary Figure 2

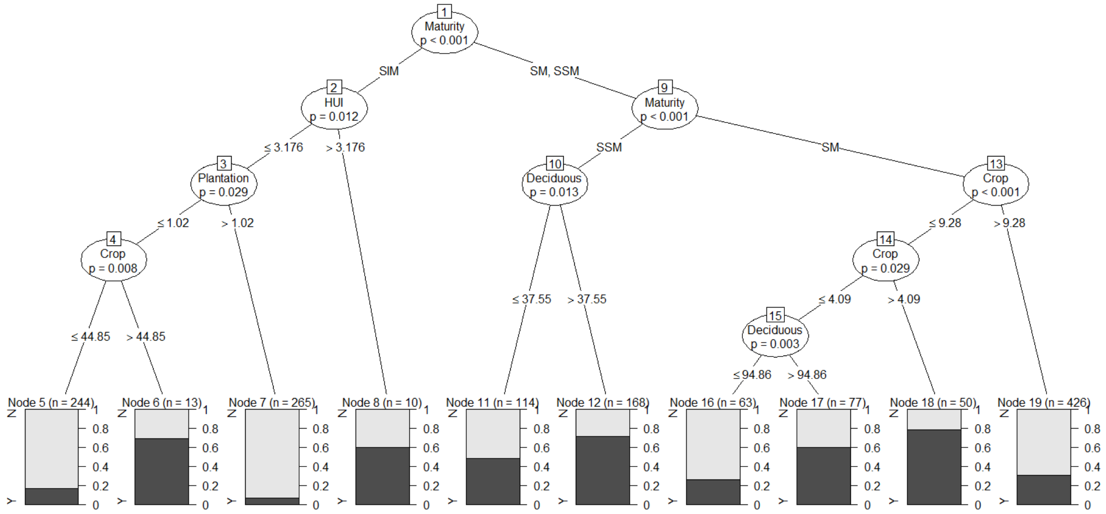

Supplementary Figure 3

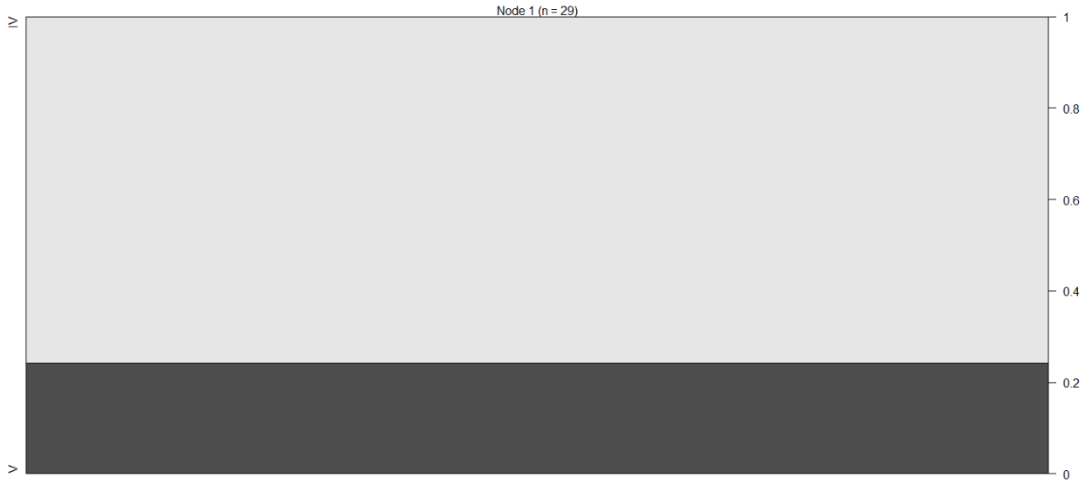

Supplementary Figure 4

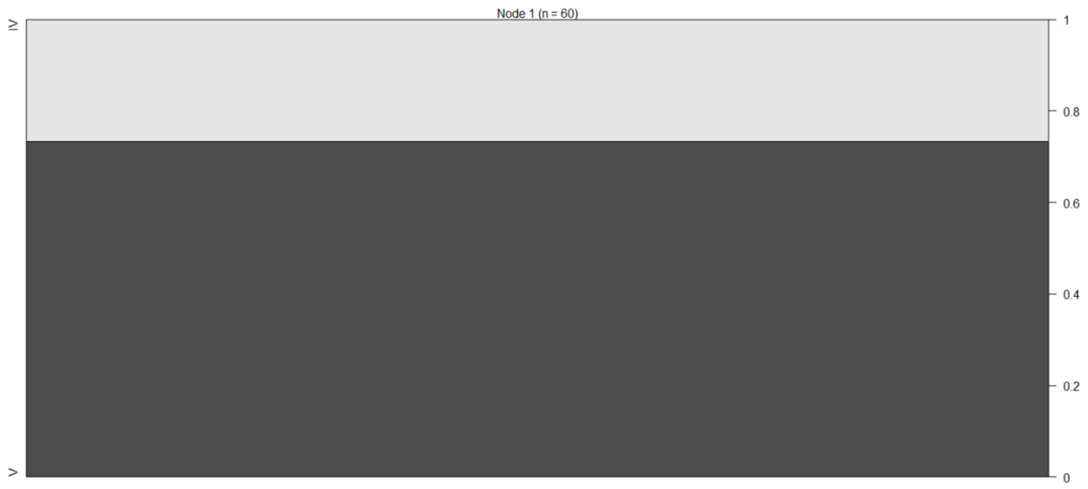

Supplementary Figure 5

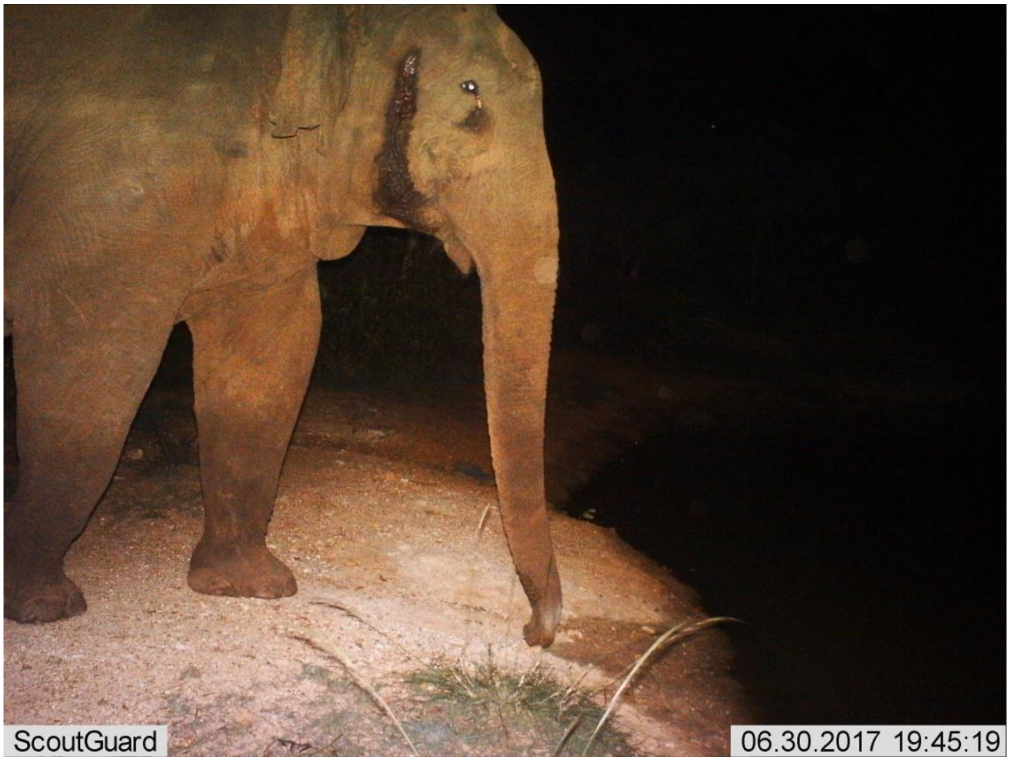

Supplementary Figure 6

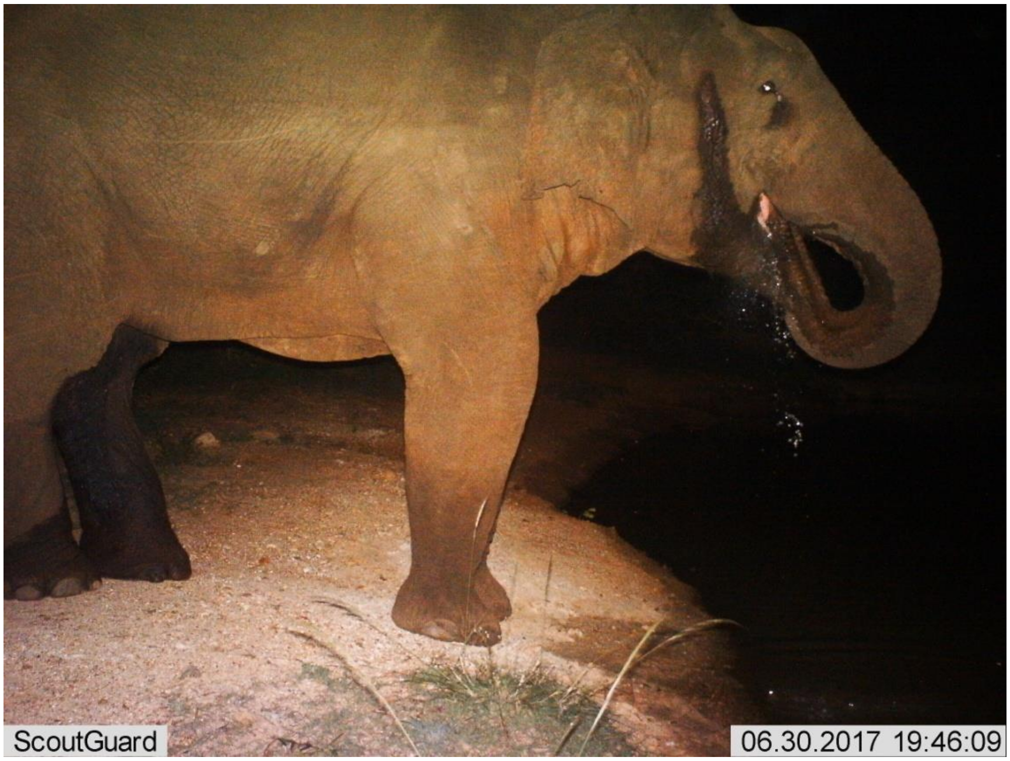

Supplementary Figure 7

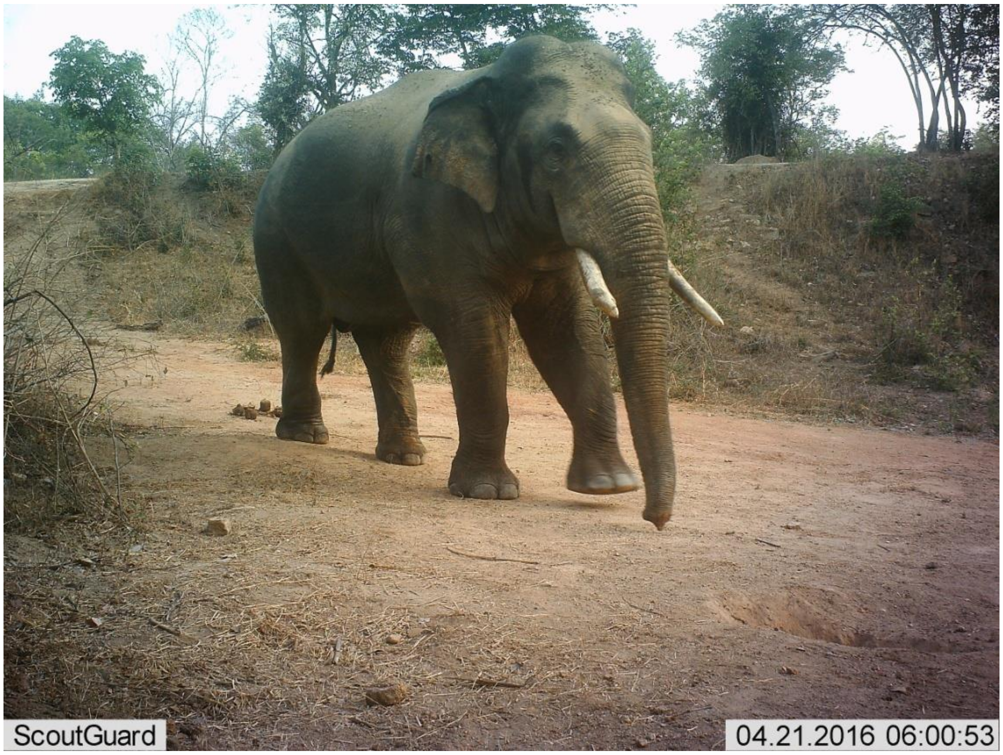

Supplementary Figure 8

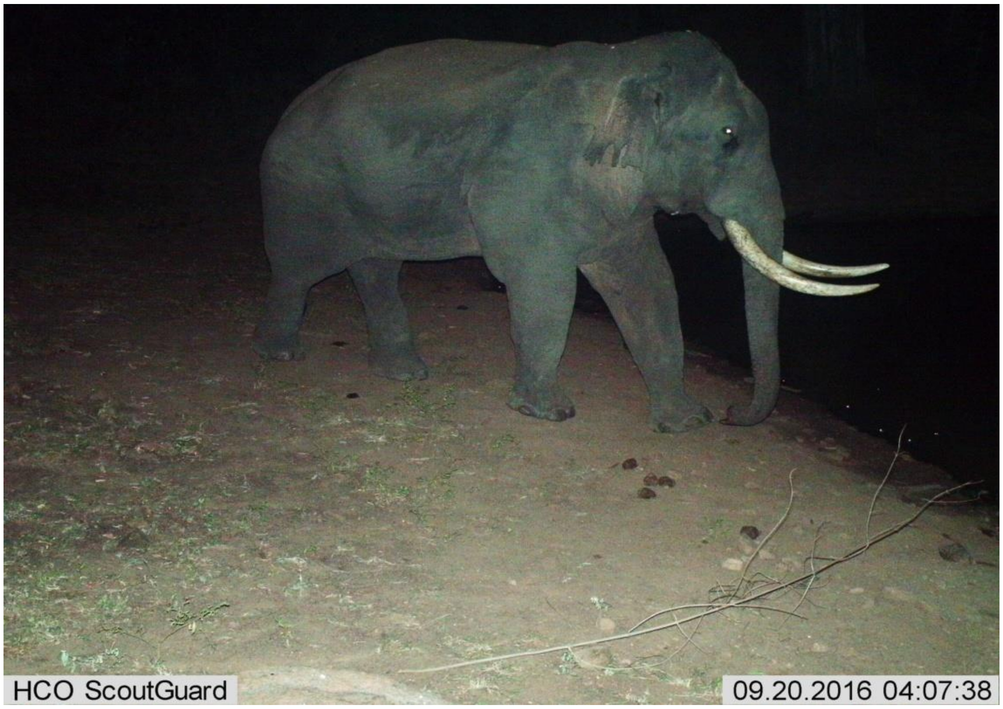

Supplementary Figure 9

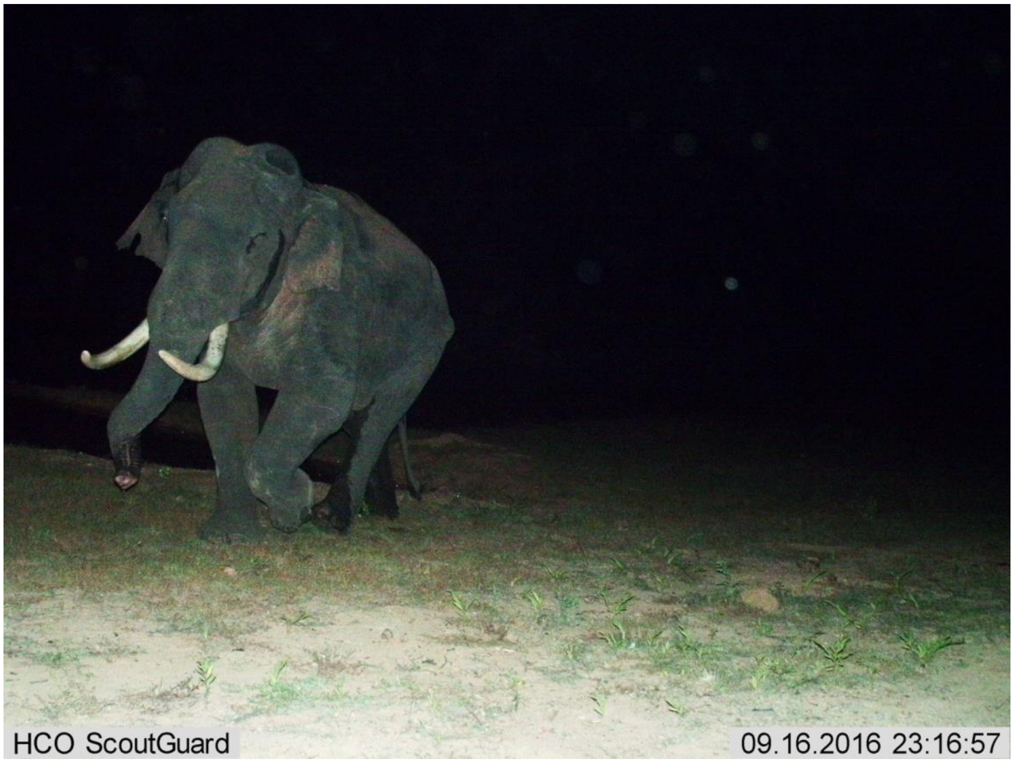

Supplementary Figure 10

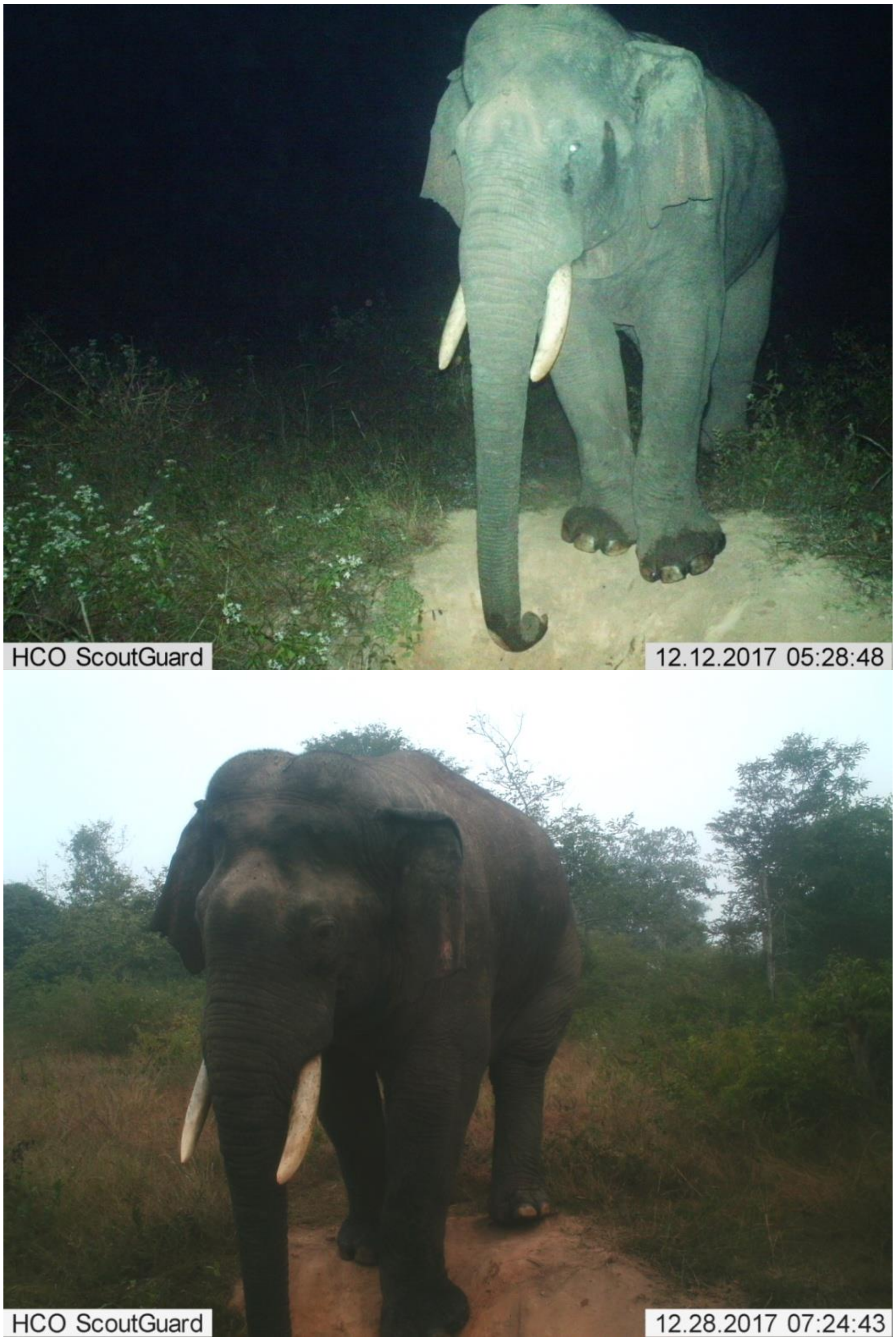

Supplementary Video 1
